# Controlled Sputtering Pressure on High-Quality Sb_2_Se_3_ Thin Film for Substrate Configurated Solar Cells

**DOI:** 10.3390/nano10030574

**Published:** 2020-03-22

**Authors:** Rong Tang, Xingye Chen, Yandi Luo, Zihang Chen, Yike Liu, Yingfen Li, Zhenghua Su, Xianghua Zhang, Ping Fan, Guangxing Liang

**Affiliations:** 1 Shenzhen Key Laboratory of Advanced Thin Films and Applications, Key Laboratory of Optoelectronic Devices and Systems of Ministry of Education and Guangdong Province, College of Physics and Optoelectronic Engineering, Shenzhen University, Shenzhen 518060, China; Rongtang.jobs@gmail.com (R.T.); ymt_0198@163.com (X.C.); lyandidi@163.com (Y.L.); chen798491170@126.com (Z.C.); fanping308@126.com (P.F.); 2 School of Material and Metallurgical Engineering, Guizhou Institute of Technology, Guiyang 550003, China; liuyikecsu@163.com (Y.L.); lyf350857423@163.com (Y.L.); 3 ISCR (Institut des Sciences Chimiques de Rennes) UMR 6226, CNRS, Univ. Rennes, 35042 Rennes, France; xiang-hua.zhang@univ-rennes1.fr

**Keywords:** thin film solar cell, Sb_2_Se_3_, magnetron sputtering, working pressure, post-selenization, substrate configuration

## Abstract

Magnetron sputtering has become an effective method in Sb_2_Se_3_ thin film photovoltaic. Research found that post-selenization treatments are essential to produce stoichiometric thin films with desired crystallinity and orientation for the sputtered Sb_2_Se_3_. However, the influence of the sputtering process on Sb_2_Se_3_ device performance has rarely been explored. In this work, the working pressure effect was thoroughly studied for the sputtered Sb_2_Se_3_ thin film solar cells. High-quality Sb_2_Se_3_ thin film was obtained when a bilayer structure was applied by sputtering the film at a high (1.5 Pa) and a low working pressure (1.0 Pa) subsequently. Such bilayer structure was found to be beneficial for both crystallization and preferred orientation of the Sb_2_Se_3_ thin film. Lastly, an interesting power conversion efficiency (PCE) of 5.5% was obtained for the champion device.

## 1. Introduction

Antimony selenide (Sb_2_Se_3_) has become a promising material for new-generation thin film solar cells as power conversion efficiency (PCE) of the device increased from 1.9% to 9.2% in only a few years [1,2,3,4]. Compared with traditional thin film solar cells such as CIGS, CdTe, CZTS, and perovskite, Sb_2_Se_3_ possesses some intrinsic advantages. First of all, the material has excellent optoelectronic properties, for instance, an ideal optical band gap (1.1–1.3 eV), outstanding absorption coefficient (>10^5^ cm^−1^), decent carrier mobility (~10 cm^2^V^−1^s^−1^), and long carrier lifetime (~60 ns), making it as a suitable light absorption layer for efficient thin film solar cells [3,4,5]. Second, unlike multielement compound thin film solar cells such as CGIS and CZTS, Sb_2_Se_3_ gets rid of complex composition control and unwanted impurity phases during the deposition and crystallization procedures, as the material has only one single phase of orthorhombic structure. Further, another major distinct advantage of Sb_2_Se_3_ over the most well-developed thin film solar cells, i.e., CIGS and CdTe, is the material is low-cost and environment-friendly since both Sb and Se are earth-abundant and low-toxicity elements. Hence, it is reasonable to suppose that Sb_2_Se_3_ technology will become a strong candidate for the mass production of thin film photovoltaic modules.

As being one of the most competitive absorber candidates for the next-generation thin film photovoltaic, Sb_2_Se_3_ has attracted much attention and thereby various deposition techniques; thus, electrochemical [6], solution [7,8,9], thermal evaporation [1,2,5,10], vapor transport deposition (VTD) [3,11,12,13], close-spaced sublimation (CSS) [4,14], and sputtering [15,16,17,18,19] have been thoroughly explored to enhance the PCE of the devices. Among these deposition approaches, magnetron sputtering is a straightforward technique that is widely used in conventional thin-film photovoltaic technologies such as CIGS and CZTS [20,21]. The method is well-known for its merits of good uniformity, full-vacuum operation, and excellent composition transfer of the target materials, although to date, for Sb_2_Se_3_ photovoltaic, devices with the highest PCEs have been fabricated via VTD and CSS [3,4]. Magnetron sputtering has been proven as an effective method to produce highly efficient Sb_2_Se_3_ solar cells by our group as well. Liang reported a decent PCE of 3.35% for sputtered Sb_2_Se_3_ followed by a in situ heating treatment [16]. Chen presented an efficient quasi-homojunction Sb_2_Se_3_ thin-film prepared using the sputtering method [17]. Very recently, a competitive PCE of 6.06% was achieved by selenizing sputtered amorphous Sb_2_Se_3_ thin-film by our group [22], suggesting a huge potential of the magnetron sputtering technique for producing efficient Sb_2_Se_3_ thin film solar cells. 

It has to be pointed out that in previous works where Sb_2_Se_3_ devices were fabricated using magnetron sputtering, efforts were mainly focused on investigations of heat treatment processes. However, the influences of sputtering parameters, for instance, sputtering power, working pressure, target-to-substrate distance, etc., on the photovoltaic performance of the devices were seldom reported. Working pressure was discovered to be an essential factor for both metal and semiconductor sputtering in thin-film solar cell fabrications [23,24]. Severe problems such as pinholes, blistering, and even delamination occur once an improper working pressure is applied. In this work, the effect of working pressure on Sb_2_Se_3_ thin-film solar cells has been thoroughly studied. By tuning the working pressure during the deposition process, an interesting PCE of 5.52% has been obtained for the champion device in substrate configuration of glass/Mo/Sb_2_Se_3_/CdS/ITO/Ag.

## 2. Experimental Details

### 2.1. Deposition of Sb_2_Se_3_ Thin Film

Sb_2_Se_3_ powder with high purity (>99.99%) was ball milled and sintered to prepare a dense Sb_2_Se_3_ sputtering target first. Mo-coated glass substrates were subsequently cleaned in an ultrasonic bath using detergent, ethanol, and deionized water, prior to sputtering deposition. The background pressure of the sputtering vacuum chamber was evacuated below 7.0 × 10^−4^ Pa after sample loading. The sputtering power and duration were fixed at 35 W and 90 min, respectively, for all samples. The working pressure was adjusted from 0.1 Pa to 2.0 Pa to investigate the effect of working pressure on device performance. Homogeneous amorphous Sb_2_Se_3_ films were obtained once the sputtering process was finished. The as-deposited samples were then transferred into a vacuum tubular furnace for post selenization treatment. A graphite container with 0.15 g of selenium powder in it was utilized to store the amorphous Sb_2_Se_3_ films. The tubular furnace was evacuated to a relatively low background pressure prior to the selenization; after that, high-purity Ar (>99.999%) was pumped into the furnace and the working pressure was kept at 5 × 10^4^ Pa during the whole annealing process. The furnace temperature was then ramped up to 350 °C at a heating rate of 20 °C/min. The selenization duration was fixed at 15 min for each sample. The furnace was naturally cooled down to room temperature after the heating program was finished. 

### 2.2. Device Fabrication

CdS buffer layer was deposited onto the crystallized Sb_2_Se_3_ films using chemical bath deposition (CBD). Magnetron sputtered ITO was deposited for 40 min with a DC power of 60 W onto the CdS layer. The device surface was scribed into small squares with identical area by knife, and Ag electrodes were deposited onto the ITO surface via thermal evaporation to form metallic contact (the active area of each device is 0.15 cm^2^). A substrate configuration of glass/Mo/Sb_2_Se_3_/CdS/ITO/Ag was assembled for our Sb_2_Se_3_ solar cells. 

### 2.3. Characterization of the Sb_2_Se_3_ Films and Devices

The surface and cross-sectional microstructures of the Sb_2_Se_3_ films were characterized using a scanning electron microscope (SEM, SUPRA 55, Zeiss, Oberkochen, Germany). X-ray diffraction (XRD, Ultima-iv, Rigaku, Tokyo, Japan, *CuK_α_* radiation under operation conditions of 40 kV and 40 mA from 10° to 60°) was utilized to investigate the crystal orientation of the Sb_2_Se_3_ films. The current density–voltage (*J–V*) curves of the Sb_2_Se_3_ devices were measured under 100 mW/cm^2^ AM 1.5 G light illumination using a class AAA solar simulator at room temperature. The external quantum efficiency (EQE) spectra were measured using a Zolix SCS101 system (Zolix Instruments, Beijing, China) and a Keithley 2400 source meter (Keithley Instruments, Solon, OH, USA).

## 3. Results and Discussions

**Figure 1:**
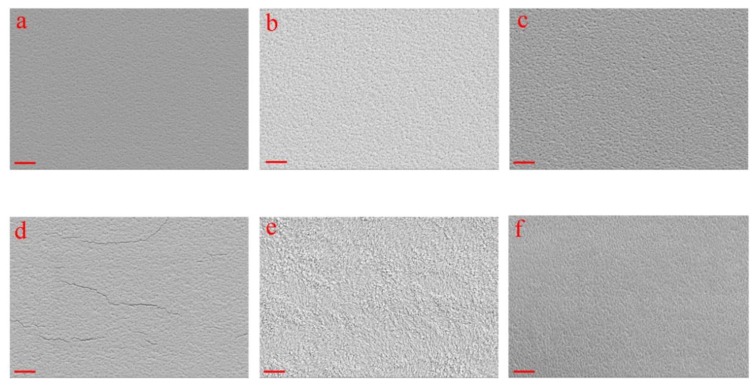
Scanning electron microscope (SEM) top-view images of as-deposited Sb_2_Se_3_ thin films sputtered under 2.0 Pa (**a**), 1.5 Pa (**b**), 1.0 Pa (**c**), 0.5 Pa (**d**), 0.1 Pa (**e**), and mixed pressures (**f**). The red scale bar at the left bottom corner of each figure reads 1 µm.

**Figure 2:**
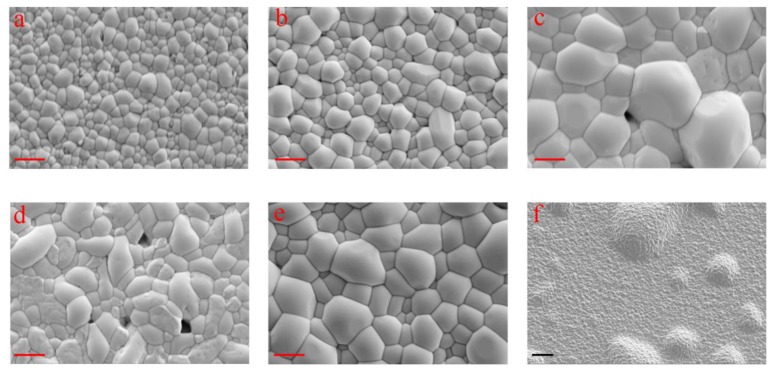
SEM top-view images of crystallized Sb_2_Se_3_ thin films sputtered under 2.0 Pa (**a**), 1.5 Pa (**b**), 1.0 Pa (**c**), 0.5 Pa (**d**), mixed pressures (**e**), and 0.1 Pa (**f**). The red scale bars at the left bottom corner of (**a**–**e**) read 1 µm. The black scale bar at the left bottom corner of (**f**) reads 10 µm.

Sb_2_Se_3_ thin films sputtered under various working pressures were denoted as 2.0-Sb_2_Se_3_, 1.5-Sb_2_Se_3_, 1.0-Sb_2_Se_3_, 0.5-Sb_2_Se_3_, and 0.1-Sb_2_Se_3_, respectively. Bilayer deposition using a mixture of two different working pressures is a common technique utilized in Mo sputtering to produce conductive Mo back contact with good adhesion [25]. Herein, in order to study the effect of mixed working pressure, a working pressure of 1.5 Pa was firstly applied for 20 min to sputter the bottom layer, then the working pressure was decreased to 1.0 Pa and the sputtering process was further carried out for 70 min to deposit the top layer. The sample was denoted as mixed-Sb_2_Se_3_ accordingly. The thicknesses of the as-deposited thin films were measured as 260 nm, 480 nm, 660 nm, 690 nm, 810 nm and 630 nm for the 2.0-Sb_2_Se_3_, 1.5-Sb_2_Se_3_, 1.0-Sb_2_Se_3_, 0.5-Sb_2_Se_3_, 0.1-Sb_2_Se_3_, and mixed-Sb_2_Se_3_, respectively, by using a stylus profilometer. SEM top-view images of the as-deposited and annealed Sb_2_Se_3_ thin films via various working pressures are displayed in Figure 1 and Figure 2, respectively. As can be seen from the figures, the as-deposited 2.0-Sb_2_Se_3_, 1.5-Sb_2_Se_3_, 1.0-Sb_2_Se_3_, and mixed-Sb_2_Se_3_ thin films showed very similar surface morphologies. No distinct crystal grain could be observed on the surfaces of these samples. However, cracks appeared on the surface of the 0.5-Sb_2_Se_3_ sample (Figure 1d). When the working pressure was low, the number of argon atoms decreased and, as a result, the probability of collision between the sputtered Sb_2_Se_3_ molecules and argon atoms decreased too, thereby leading to an excessive rate of the thin film deposition. Such rapid deposition might enhance the defect density and stress within the as-deposited Sb_2_Se_3_ thin films, which might induce crack formation within the bulk film. Once the working pressure was too low, i.e., 0.1 Pa, a slightly different surface morphology could be observed for the 0.1-Sb_2_Se_3_ sample in Figure 1e where the thin film surface seemed to be getting rougher and irregular, possibly due to partial crystallization of the deposited film caused by the ultrahigh energy. 

For the annealed samples, it is apparent that the grain size of the Sb_2_Se_3_ thin film increased when the working pressure was decreased from 2.0 Pa to 1.0 Pa, as shown in Figure 2a–c. The change could be attributed to the elevated energies of the sputtered Sb_2_Se_3_ molecules under low working pressures, which would facilitate the crystallization of the thin films. Pinholes appeared on the surfaces of the 1.0-Sb_2_Se_3_ and 0.5-Sb_2_Se_3_ samples (Figure 2c,d), which is probably due to the higher defect density and stresses within the amorphous films caused by overly high deposition rates. Significant blistering was clearly observed for the 0.1-Sb_2_Se_3_ sample (Figure 2f), possibly owing to the sudden release of the excessive compressive stress during the selenization process. Interestingly, a pinhole-free surface consisted of large crystal grains could be seen for the mixed-Sb_2_Se_3_ sample, as shown in Figure 2e. We attribute this optimal morphology to the bilayer structure of the sample, where the lattice mismatch between the Mo substrate and the Sb_2_Se_3_ thin film could be suppressed by the Sb_2_Se_3_ bottom layer sputtered at 1.5 Pa, whilst the Sb_2_Se_3_ top layer sputtered at 1.0 Pa contributed to the crystallization promotion. 

**Figure 3:**
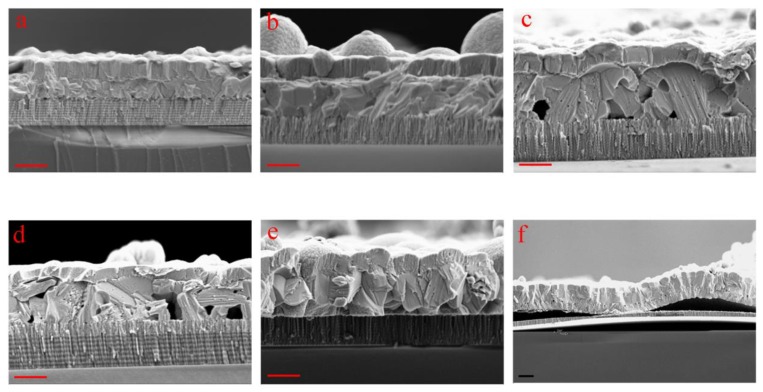
SEM cross-sectional images of Sb_2_Se_3_ devices sputtered under 2.0 Pa (**a**), 1.5 Pa (**b**), 1.0 Pa (**c**), 0.5 Pa (**d**), mixed pressures (**e**), and 0.1 Pa (**f**). The red scale bars at the left bottom corner of (**a**–**e**) read 1 µm. The black scale bar at the left bottom corner of (**f**) reads 2 µm.

Figure 3 demonstrates the cross-sectional morphologies of the Sb_2_Se_3_ devices sputtered under different working pressures. As can be seen from the figures, sputtering the Sb_2_Se_3_ thin films at high working pressures, e.g., 2.0 Pa and 1.5 Pa, would produce compact crystal grains for the samples. On the other hand, the thin film thicknesses were hindered owing to slow deposition rates at high working pressures, restricting the sizes of the Sb_2_Se_3_ grains. Pinholes at the Sb_2_Se_3_/Mo junction are clearly seen for those samples sputtered at 1.0 Pa and 0.5 Pa, consistent with the SEM top-view images. It is also noted that an overly thick MoSe_2_ interfacial layer could be clearly observed between the Mo substrate and the Sb_2_Se_3_ thin film for these two samples. We speculate that the pinholes within these samples would act as tunnels to accelerate the diffusion of Se vapor towards the Sb_2_Se_3_/Mo interface, inducing the formation of the thick MoSe_2_ interfacial layer during the selenization treatment. Large vertically-oriented Sb_2_Se_3_ grains without visible pinholes are observed for the annealed mixed-Sb_2_Se_3_ sample. No apparent bilayer structure could be seen within the bulk film, suggesting excellent grain continuity across the whole film after crystallization. Figure 3f displays the cross-sectional image of the 0.1-Sb_2_Se_3_ sample. Complete delamination between the Sb_2_Se_3_ absorber layer and the Mo back contact is clearly observed due to the blistering effect, which would definitely lead to device failure.

**Figure 4:**
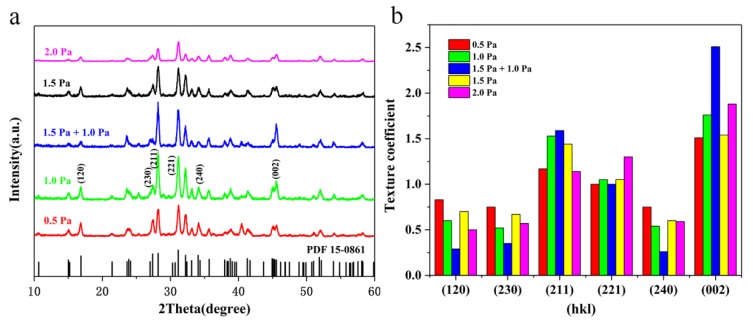
Morphology evolutions of Sb_2_Se_3_ thin films. XRD data of crystallized Sb_2_Se_3_ thin films (**a**). Texture coefficients (*TC*) of crystallized Sb_2_Se_3_ thin films (**b**).

It is well known that film orientation plays as an essential role in the photovoltaic performance of Sb_2_Se_3_ thin film solar cells. Reports have shown that transport of photogenerated carriers would be facilitated within the bulk film once the Sb_2_Se_3_ grains are in vertical orientations, as the carriers could travel readily within the covalently bonded ribbons in this case. On the other hand, the photogenerated carriers would have to hop between the Sb_2_Se_3_ ribbons held together by van der Walls forces when the Sb_2_Se_3_ grains are in improper orientations (hk0), making the carrier transport difficult [2,3,4,5]. Therefore, it is vital to optimize the film orientation in order to obtain better device performance. XRD results of the annealed Sb_2_Se_3_ thin films at various working pressures are illustrated in Figure 4a. All the samples demonstrate vertical orientations, as evidenced by the prominent diffraction peaks of (211) and (221). Texture coefficients (*TC*) of the diffraction peaks were calculated using the equation below to quantitatively investigate the orientation preference (Figure 4b) [26]:

(1) $$TChkl=\frac{I(hkl)}{I0(hkl)}/(\frac{1}{N}\sum_{i=1}^{N}\frac{I(h_{i}k_{i}l_{i})}{I0(h_{i}k_{i}l_{i})})$$

where *I*_(*hkl*)_ and *I*_0(*hkl*)_ are the diffraction peak intensities of (*hkl*) planes in the measured and standard XRD pattern of Sb_2_Se_3_ (JCPDS 15-0861), respectively. Large *TC* value of a diffraction peak indicates preferred orientation along this particular direction [26]. As can be seen from the figure, the mixed-Sb_2_Se_3_ sample shows the highest overall *TC* values for the (*hkl*, l≠0) planes and the lowest *TC* values for the (*hk*0) planes among all the samples, indicating the optimal growth orientation of the film. Such preferential vertically oriented Sb_2_Se_3_ grains significantly improved carrier transport within the film and thus enhance the device performance. In contrast, the *TC* values of the (*hk*0) planes, namely, (120), (230), and (240), were much larger for the other samples, leading to device deterioration due to high series resistance [2].

**Figure 5:**
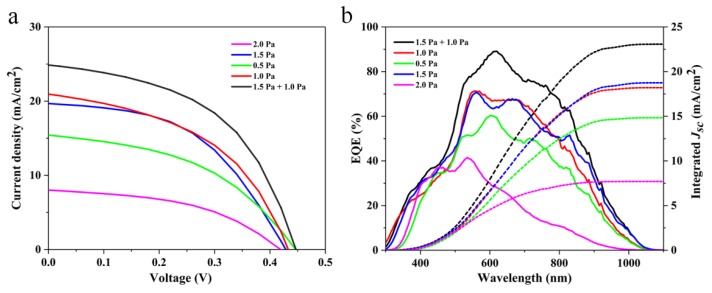
Device performances. Current density–voltage (*J–V*) curves (**a**), and external quantum efficiency (EQE) and integrated *J_SC_* (**b**) of Sb_2_Se_3_ devices fabricated under various working pressures.

The current density–voltage (*J–V*) curves, external quantum efficiency (EQE), and detailed performance parameters of the Sb_2_Se_3_ devices prepared at various working pressures are summarized in Figure 5 and Table 1. The champion mixed-Sb_2_Se_3_ device offered a short-circuit current density (*J_SC_*) of 24.95 mA/cm^2^, an open circuit voltage (*V_OC_*) of 448 mV, and a fill factor (*FF*) of 53.2%, thus achieving an interesting PCE of 5.5%. It can be seen that although other devices presented decent *V_OC_* values, the final PCEs of these devices were restricted by the low *FF* and especially the poor *J_SC_*, which were far inferior to that of the champion mixed-Sb_2_Se_3_ device. We attribute the unsatisfied *J_SC_* of the devices sputtered at low working pressures (below 1 Pa) to the shunt paths caused by the pinholes within the films, whilst the low *J_SC_* of the devices sputtered at elevated working pressures (above 1.5 Pa) could be attributed to the insufficiently thick absorber layer at such high working pressures, as shown in SEM images. In addition, the undesired grain orientations of other films (as already demonstrated quantitatively by the XRD results) prepared under improper working pressures would increase the series resistance of the devices and thus lead to poor *FF* and *J_SC_* [2]. In order to analyze the photoresponses of the devices fabricated under various working pressures, EQE spectra were measured for all the devices. Apparently, the EQE curve of the mixed-Sb_2_Se_3_ device was much higher than that of the other samples in the wavelength region from 500 nm to 850 nm, suggesting less recombination losses of photogenerated carriers not only within the bulk film but also at the Sb_2_Se_3_/CdS heterojunction [3].

## 4. Conclusions

In summary, the influence of working pressure on the device performance of sputtered Sb_2_Se_3_ thin film solar cells has been investigated in this work. High-quality Sb_2_Se_3_ thin film was obtained when a bilayer structure was applied by sputtering the film at a high (1.5 Pa) and a low working pressure (1.0 Pa) subsequently. Such bilayer structure was found to be beneficial for both crystallization and preferred orientation of the Sb_2_Se_3_ thin film. Further, shunt leakages induced by pinholes within the bulk film could be effectively hindered by using the bilayer structure. Finally, an interesting PCE of 5.5% was obtained for the champion device. 

## Figures and Tables

**Table 1:** Device performance parameters of the Sb_2_Se_3_ devices prepared at various working pressures.

Samples	*V_oc_* (mV)	*J_sc_* (mA/cm^2^)	*FF* (%)	*E_ff_* (%)
2.0-Sb_2_Se_3_	408	8.76	43.1	1.51
1.5-Sb_2_Se_3_	430	19.64	49.7	4.10
1.0-Sb_2_Se_3_	434	21.01	48.4	4.22
0.5-Sb_2_Se_3_	448	15.44	45.7	3.10
mixed-Sb_2_Se_3_	448	24.95	53.2	5.52
